# ‘Nurses’ Assessment and Perception of Live Music in the Intensive Care Unit: A Convergent Mixed‐Methods Study

**DOI:** 10.1111/nicc.70356

**Published:** 2026-02-09

**Authors:** Linette Thorn, Anna Holm, Trine Højfeldt Lund, Margrethe Langer Bro, Pia Dreyer

**Affiliations:** ^1^ Department of Intensive Care Aarhus University Hospital Aarhus Denmark; ^2^ The Danish National Academy of Music Esbjerg Denmark; ^3^ The Royal Academy of Music Aarhus Denmark; ^4^ Department of Public Health, Department of Science in Nursing Aarhus University Aarhus Denmark

**Keywords:** critical care nursing, mixed‐methods study, music intervention, nurse well‐being, patient‐centred care

## Abstract

**Background:**

Live music interventions in hospital settings have been shown to alleviate anxiety, reduce pain and enhance patient well‐being—not only for patients but also for healthcare professionals working in high‐stress environments such as intensive care units (ICUs). However, limited research has explored ICU nurses' experiences of live music.

**Aim:**

The aim of this study was to describe nurses' personal and professional assessment and perception of patient‐tailored live music in the ICU.

**Study Design:**

A convergent mixed‐methods study was conducted at a Danish university hospital. Quantitative data were collected via a content‐validated questionnaire among ICU nurses (*n* = 221; response rate 72%), while qualitative data were obtained through semistructured interviews with a purposive sample of nurses (*n* = 7). Data were analysed separately and merged during interpretation.

**Results:**

Nurses perceived live music as a valuable, emotionally resonant and professionally enriching intervention. Questionnaire results showed high levels of agreement that live music was meaningful (*M* = 1.56, SD = 0.75) and a positive experience (*M* = 1.39, SD = 0.59). The qualitative survey responses and interview data were analysed into three main categories and four subcategories: (1) Nurses' personal experience—*an emotional breath of fresh air* and *an intense and meaningful atmosphere*; (2) nurses' professional experience—*bringing people together* and *bringing spirit, energy and motivation into care*; and (3) the nurse as a gatekeeper shielding the patient.

**Conclusions:**

Live music in the ICU appears to benefit not only patients but also nurses, by supporting emotional well‐being and enhancing the quality of care. It may serve as a holistic intervention that strengthens both the personal and professional dimensions of nursing practice.

**Relevance to Clinical Practice:**

Live music is a feasible and meaningful addition to ICU care. Nurses' active involvement is essential to ensure interventions remain sensitive to patient needs and clinical context. Collaboration with specially trained professional musicians may further enhance effective and sustainable implementation.

## Impact Statements

- What is known about the topic
  ○ Live music can reduce anxiety, pain and stress in hospital patients.
  ○ Live music is a feasible and accepted intervention in ICU settings.
  ○ Limited research exists on ICU nurses' experiences with live music.
- What this paper adds
  ○ Live music supports ICU nurses' emotional well‐being and professional pride.
  ○ Live music fosters presence, human connection and holistic care.
  ○ Nurses act as compassionate gatekeepers to protect vulnerable patients.

## 1 Introduction

A growing body of research demonstrates that music interventions in hospital settings can alleviate anxiety and perceived pain, promote relaxation and improve patient outcomes [1]. A report by the World Health Organization (WHO) synthesises global evidence on the role of the arts, including music, in improving health and well‐being [2].

Whether through patient‐tailored live music interventions or curated playlists, the benefits of music in healthcare are increasingly acknowledged, making it a valuable tool for enhancing patient care and overall well‐being [1]. In the ICU setting, live music has been identified as an acceptable, appropriate and feasible non‐pharmacological intervention [3, 4, 5]. The positive impacts of music may also be beneficial to healthcare professionals working in high‐pressure ICU environments.

## 2 Background

ICU nurses face numerous challenges that significantly impact their well‐being. The high‐stakes environment, where decisions can mean the difference between life and death, creates intense pressure. They often manage complex patient cases, navigate family dynamics and confront ethical dilemmas—all while maintaining a compassionate demeanour [6]. The emotional burden of caring for critically ill or terminal patients can lead to compassion fatigue and burnout [7]. Additionally, the fast‐paced nature of the ICU, combined with staff shortages and high patient turnover, can contribute to feelings of being overwhelmed and exhausted [8]. For these reasons, nurses are considered a professional group at high risk of experiencing work‐related stress [8]. Therefore, organisational strategies aimed at preventing burnout and enhancing nurses' job satisfaction could improve the quality of patient care [9]. This is essential to maintain nurses' motivation and commitment to their work [10]. One study found that nurses derive meaning from their work through their commitment to patient care and their desire to make a positive impact on patients' lives [11]. This enduring motivation highlights one of the core values driving nursing practice, namely the profession's deep connection to patient well‐being and the fulfilment derived from making a difference to others [12].

Patient‐centred care (PCC) is a major component of high‐quality care. It involves a whole‐person perspective and is grounded in respect for patients' values, needs and preferences, while also informing, educating and involving them in decision‐making, and providing emotional support [13]. Smilde et al. found that patient‐tailored live music interventions in surgical wards may contribute to PCC [14]. PCC may have a positive impact on nursing by improving job satisfaction, self‐confidence and quality of care and reducing stress and burnout among healthcare professionals [15]. Berg et al. found that live music in medical wards supports nurses in delivering compassionate care to hospitalised patients. It significantly enhanced patient engagement and fostered a sense of connection. Music has the power to evoke emotions, reduce stress and create a calming environment, making it an effective tool in person‐centred, compassionate care [16]. Furthermore, Bro et al. [17] found that patient‐tailored live music interventions during haemodialysis created a relaxing and de‐stressing experience for the nurses. Additionally, these interventions positively impacted nurses' work engagement by enhancing patient relationships and fostering compassion [18]. While several studies have explored the effects of music on patients and the feasibility of music interventions in ICUs, little is known about how these interventions are perceived by the nurses who observe, facilitate and respond to them. This represents a critical gap in our understanding of music's role in holistic ICU care. Given ICU nurses' central role in patient‐centred care and their exposure to emotionally intense clinical settings, their perspectives offer valuable insight into both caregiver well‐being and the broader therapeutic context of live music. Exploring their experiences can thus inform the development of meaningful, context‐sensitive interventions.

## 3 Aim

The present study aimed to describe nurses' personal and professional assessments and perceptions of patient‐tailored live music in the ICU by integrating quantitative and qualitative findings.

## 4 Study Design

### 4.1 Design and Methods

**FIGURE 1:**
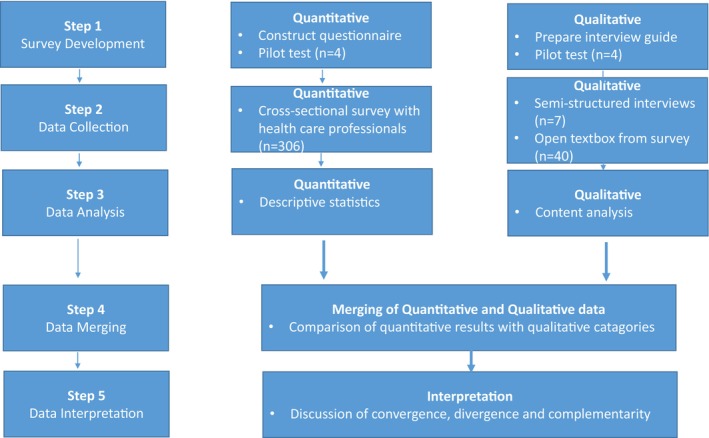
Flow chart of convergent mixed‐methods research design.

We applied a convergent mixed‐methods approach as it allows for a richer, more nuanced understanding of the impact of live music on ICU nurses by combining quantitative trends with qualitative depth [19]. The mixed‐methods design is illustrated in Figure 1. To ensure clear, transparent and rigorous reporting, we used the Good Reporting of A Mixed Methods Study (GRAMMS) framework [20].

### 4.2 The Music Intervention

Prior to the project, participating music students from a Danish music academy completed a standardised training programme. The programme's framework, artistic approach and practical components—including procedures for patient interaction and collaboration with staff—are described in detail in a study by Bro et al. [21]. Each music intervention consisted of three components: (1) a briefing by the nurse on the patient's physical and mental condition, (2) patient‐tailored live music in the patient's room and (3) debriefing between the musician and nurse.

During the session, the musician interacted with the patient through continuous non‐verbal observation, including monitoring breathing patterns, facial expressions, body tension and overall responsiveness. Based on these cues, the musician intuitively selected and adjusted the repertoire, tempo, intensity and musical expression to match the patient's immediate condition while maintaining artistic integrity. Tailoring was, therefore, dynamic and responsive, guided by both the preliminary nurse briefing and the patient's immediate reactions. Adjustments could further be modulated by the presence of relatives in the room. Each intervention lasted approximately 10–20 min and was offered once per week.

### 4.3 Setting and Participants

The study was conducted at the ICU department of a university hospital in Denmark, which consisted of five units with a total of 42 beds, receiving approximately 3500 patients annually from all medical and surgical specialties. The department maintained a 1:1 nurse‐to‐patient ratio and had an open visiting policy for relatives. Relatives were welcome to be present during the music intervention.

At the time of the study, 306 nurses were employed in the ICUs. The inclusion criteria were all clinical ICU nurses employed during the study period, while nurses on maternity or long‐term sick leave were excluded. All eligible nurses were invited to participate in the survey. For the interviews, a purposive sampling strategy was employed to recruit nurses from different ICUs, ensuring variation in experiences and perspectives.

### 4.4 Data Collection

Data were collected between May and September 2023.

Quantitative data were collected via a purpose‐designed questionnaire. The questionnaire was divided into two sections: (1) the nurses’ personal experience of live music and (2) their professional experience as a healthcare provider. Responses included multiple‐choice items and statements rated on a 5‐point interval Likert scale ranging from ‘strongly agree’ to ‘strongly disagree’. In total, the questionnaire consisted of 10 Likert‐scale items plus demographic questions (age, gender, years of ICU experience and experience with live music interventions). At the end, the questionnaire included an open text box for additional comments.

The questionnaire underwent face and content validation by four ICU nurses with expertise in clinical practice, research and leadership. They assessed the questionnaire's logic, clarity and relevance [22]. The online questionnaire was created using SurveyXact [23] and distributed via e‐mail to all eligible nurses. Three reminders were sent to non‐responders.

Qualitative data were collected through semistructured interviews with ICU nurses. The interviews were based on Brinkmann and Kvale's method, which also informed the development of the interview guide [24]. The interview guide was validated by four nurses from the research team and pilot tested. Questionnaire items informed the structure of the interview guide. Interviews were conducted face to face in a single room and subsequently transcribed.

### 4.5 Data Analysis

In line with the convergent mixed‐methods design, quantitative and qualitative data were analysed separately and later integrated during interpretation in the Results section [19].

Descriptive statistical analyses of the survey data (means, standard deviations and frequencies) were conducted using Stata/MP 18.0 [25]. Normality was assessed visually using histograms. As the study was exploratory, no formal tests of normality or inferential statistical tests were performed.

Qualitative data, including open‐text survey responses and interview transcripts, were analysed using inductive qualitative content analysis. The analysis was guided by the overall research questions, focusing on nurses' personal and professional experiences. The inductive analysis involved three steps: (1) familiarisation with the data, during which the first author thoroughly read the material to gain an in‐depth understanding; (2) open coding, where data were coded without predefined categories; (3) categorisation, where patterns in the data were grouped to reflect shared experiences and insights [26]. This process was conducted in NVivo [27].

All coding was conducted by the first author and subsequently reviewed by two co‐authors. Disagreements were discussed until consensus was reached. To strengthen trustworthiness, we addressed credibility (triangulation of questionnaire and interview data), transferability (rich contextual descriptions and quotations), dependability (audit trail in NVivo and peer review of coding) and confirmability (reflexive team discussions). The first author is an ICU nurse, which provided insider knowledge but also a potential source of bias. This was mitigated by collaboration with co‐authors from diverse professional backgrounds.

The development of categories from meaning units, codes and subcategories is presented in Table 1.

**TABLE 1:** Development of categories from meaning units, codes and subcategories.

Example meaning unit (text)	Codes	Subcategory	Category
*‘I had to take my glasses off… it was so moving.’ (Informant 6)* *‘A small breath of fresh air… reflections on how fragile life is.’ (Respondent 19)* *‘I let go of professionalism… the tears are streaming.’ (Respondent 37)*	Emotional release; being moved; overwhelm; reflection	An emotional breath of fresh air	Nurses' personal experience
*‘We are not just nurses – we are also humans.’ (Informant 7)* *‘A beautiful moment—a treat for both patient and me.’ (Informant 3)* *‘The room is filled with presence and meaningful atmosphere.’ (Respondent 10)*	Presence; intimacy; calm; meaningful moment	An intense and meaningful atmosphere	
*‘Music brings people together… It unites.’ (Informant 2)* *‘Brings the family together… a space not defined by illness.’ (Informant 3)* *‘It gives motivation and gathers the family.’ (Respondent 26)* *‘I felt fortunate to share such a beautiful moment.’ (Respondent 28)*	Togetherness; unity; shared emotional space	Bringing people together	Nurses' professional experience
*‘I become less stressed.’ (Informant 1)* *‘Nice to know music is coming – a plus in the book’ (Informant 4)* *‘My mind gets a break… my brain gets peace.’ (Respondent 21)* *‘Enhances quality of nursing—addressing the whole person.’ (Informant 7)*	Stress reduction; mental break; renewed energy; professional pride	Bringing spirit, energy and motivation into care	
*‘Sometimes we say no because patients cannot cope.’ (Informant 1)* *‘Some should not have too many stimuli.’ (Informant 3)* *‘Some patients are too tired… would rather sleep.’ (Respondent 20)* *‘Difficult to judge when neither patient nor relatives can be asked.’ (Respondent 2)*	Protecting patients; overstimulation; fatigue; uncertainty	—	The nurse as a gatekeeper shielding the patient

In the final stage, quantitative survey responses and qualitative data, including interviews and open‐text survey comments, were merged and interpreted jointly. The results section first presents the quantitative findings, complemented by illustrative quotes from the qualitative analysis to provide contextual depth. The integration of quantitative and qualitative results can be seen in the joint display in Table 4.

### 4.6 Ethical and Institutional Approvals

According to Danish law and The Danish Healthcare Act § 42, interviews and survey studies of this kind require no formal ethical approval. The department heads of the participating ICUs approved the study in March 2023. Participation in the survey was considered to imply consent. All participants provided both oral and written consent. Participants were informed of the voluntary nature of their participation, confidentiality measures and their right to withdraw at any time.

The music intervention was considered non‐harmful, and potential overstimulation was prevented by the nurses' clinical gatekeeping role, as they could decline music on behalf of patients when deemed inappropriate. The study was conducted in accordance with the Declaration of Helsinki [28].

## 5 Results

### 5.1 Sample

Out of the 306 nurses, 221 responded to the questionnaire, which yields a response rate of 72%. Seven nurses from four different ICUs participated in the interviews; these lasted between nine and 25 min with a median length of 18 min. Demographic characteristics of both survey respondents and interview participants are presented in Table 2.

**TABLE 2:** Demographic characteristics of survey and interview participants.

Characteristics	Survey respondents (*n* = 221); named R in quotes	Interview participants (*n* = 7); named I in quotes
Age (median, range)	41 (23–67)	45 (26–64)
Gender *n* (%)	Male: 9 (4) Female: 211 (95) Do not wish to disclose: 3 (1)	Male: 1 (14) Female: 6 (86)
Did you experience live music in your department? *n* (%)	Yes: 189 (85) No: 33 (15)	—
How many times did you experience live music? *n* (%)	1 time: 18 (10) 2–3 times: 55 (29) 4–5 times: 53 (28) 6–7 times: 24 (12) 8–9 times: 13 (7) > 10 times: 26 (14)	—
ICU experience, years (median, range)	—	14 (3–28)

### 5.2 Nurses' Assessment of Patient‐Tailored Live Music

Among the 221 nurses who responded to the questionnaire, 189 had experienced live music in their department. These 189 respondents answered questions about their personal and professional experience of live music. The responses are shown in Table 3.

**TABLE 3:** The respondents' answers to the questionnaire (*n* = 189).

Nurses' personal experience of live music in the ICU	
Question	Mean (SD)
Live music is meaningful to me personally	1.56 (0.75)
Live music in the ICU is a positive experience for me	1.39 (0.59)
Live music in the ICU enhances my job satisfaction	1.90 (0.86)
Live music in the ICU improves my workday	1.91 (0.84)

Nurses' professional experience of live music in the ICU	
Question	Mean (SD)
Live music in the ICU is meaningful to my nursing care	1.82 (0.79)
Live music in the ICU is a quality improvement in nursing care	1.74 (0.79)
Live music creates meaningful moments for the patient	1.39 (0.58)
Live music has a positive impact on my relationship with the patient	2.37 (0.82)
Live music creates meaningful moments between the patient and their relatives	1.66 (0.74)

*Note:* Questions based on a 5‐point interval Likert scale: 1 = Strongly agree, 2 = Agree, 3 = Neither agree nor disagree, 4 = Disagree, 5 = Strongly disagree.

All items yielded a mean below 2, except for the statement *‘Live music has a positive impact on my relationship with the patient’* (*M* = 2.37, SD = 0.82), which indicates that the respondents either agreed or strongly agreed. This is supported by the low standard deviations across items, reflecting limited variability and consistent responses.

Two questions showed particularly low mean scores. These were *‘Live music in the ICU is a positive experience for me’* (*M* = 1.39, SD = 0.59) and *‘Live music creates meaningful moments for the patient’* (*M* = 1.39, SD = 0.58).

### 5.3 Nurses' Perception of Patient‐Tailored Live Music

The qualitative survey responses and interview data were analysed into three main categories and four subcategories. The quantitative results corresponding with the categories are presented below and qualitative findings are used to elaborate for a more comprehensive understanding.

### 5.4 The Nurses' Personal Experience of Live Music

#### 5.4.1 An Emotional Breath of Fresh Air

The results suggested that live music can serve as a supportive intervention that may help ease some of the challenges of daily clinical routines and contribute to the nurses’ emotional well‐being. The survey data clearly showed strong agreement with the statement ‘Live music is meaningful to me personally’ (*M* = 1.56, SD = 0.75). The qualitative data described that live music resonated deeply on an emotional level with nurses working in high‐pressure environments. The quotes suggested that music provided moments of personal reflection and an emotional release. A seemingly simple musical interlude could provoke a spontaneous, emotional reaction. The following quote demonstrates how music dissolved professional barriers, enabling genuine vulnerability even among those accustomed to high‐stress clinical situations: ‘It also sometimes brings out strong emotions, even in myself. At one point, I had to take my glasses off and sit there sniffling a bit because I just found it so moving. It really does something good.’ (*Informant 6*).

This sentiment was echoed in several survey responses. Respondent 19 described live music as ‘a small breath of fresh air’ amidst the demands of acute care, suggesting that music created a reflective space where both the joys and sorrows of life may be contemplated: ‘I experience live music as a small breath of fresh air in a daily life marked by acute and critical illness. It provides an opportunity for thoughts and reflections on life, both the good and the bad, and how fragile it is.’ (Respondent 19). In a similar vein, one nurse reflected on how the absence of patients and relatives in the staff room permitted a temporary shedding of professionalism, which facilitated an authentic emotional response: ‘Hearing live music at work affects me very emotionally, especially in the staff room, where I can let go of some of my professionalism since no patients or relatives are present. I have been surprised by how strongly I reacted (tears streaming)’ (*Respondent 37*).

#### 5.4.2 An Intense and Meaningful Atmosphere

Live music created a meaningful and calming atmosphere, offering nurses a sense of presence, gratitude and human connection beyond their clinical roles, which was reflected in the questionnaire item ‘Live music in the ICU is a positive experience for me’ (*M* = 1.39, SD = 0.59). In the interviews, nurses described how the music generated a meaningful, calm and peaceful atmosphere, allowing them to feel like whole human beings: One nurse encapsulated this sentiment by stating: ‘We are not just nurses – we are also humans. We have a profession, but we are also people who simply enjoy the music. It provides a meaningful escape from everything related to illness and suffering.’ (*Informant 7*). This quote highlights how music offered a temporary reprieve from the clinical pressures, allowing nurses to reconnect with their fundamental human nature.

Furthermore, the music was described as creating intimate moments that fostered a profound sense of presence: ‘The room is filled with presence and a very intense and meaningful atmosphere.’ (*Respondent 10*) In addition, the nurses expressed deep gratitude for the opportunity to experience such moments: ‘I experience it as a beautiful moment – a treat for both the patient and myself as a person and as a nurse.’ (*Informant 3*).

### 5.5 The Nurse's Professional Experience of Live Music

#### 5.5.1 Bringing People Together in a Break From Sickness and Illness

The analysis showed how music facilitated emotional release and acted as a universal language that united and connected people. This was reported in the survey item ‘Live music creates meaningful moments between the patient and their relatives’ (*M* = 1.66, SD = 0.74). In the qualitative data, nurses described how live music offered them a sense of calm and mental break from their demanding task, helping them reconnect with themselves amidst the stress of patient care: ‘That's what music can do – it brings people together across all ages and all kinds of differences. It touches the emotions that are fundamental to all humans – no matter where you come from or where you are. It truly unites!’ (*Informant 2*). Nurses were especially moved by how music brought families together during moments of crisis: ‘Music brings the family together and creates a setting, a space, that has nothing to do with illness. Music shifts the focus away from everything related to sickness and the hospital, instead creating a sense of freedom and a much‐needed break’ (*Informant 3*). In life‐threatening situations, music was described as bringing nurse and patient together in a deeply intimate and moving way: ‘I was deeply moved, partly because earlier that day the patient was close to death, and now he was lying there, absorbing it all. I felt overwhelmed in a good way and incredibly fortunate to be allowed to share such a beautiful moment with the patient’ (*respondent 28*). Qualitative findings suggest that the music could transform a tense clinical environment into a shared human experience, momentarily dissolving professional boundaries and allowing nurse and patient to connect on an emotional level.

#### 5.5.2 Bringing Spirit, Energy and Motivation Into Care

Live music was regarded as more than just a pleasant addition. It was perceived as an integral component of holistic care. By incorporating music into their practice, nurses felt better equipped to address both the physical and emotional needs of their patients, ultimately creating a more compassionate and effective care environment. This perception was reflected in survey responses to items such as ‘Live music in the ICU improves my workday’ (*M* = 1.91, SD = 0.84) and ‘Live music in the ICU is a quality improvement in nursing care’ (*M* = 1.74, SD = 0.79). In the qualitative findings, nurses emphasised that the music helped reduce their stress levels: ‘I become less stressed’ (*Informant 1*). This was elaborated in the following quote, describing how live music provided a sense of calm and brief mental break from clinical demands: ‘I find that live music is calming. In a way, I often feel stressed. The music does something good for my mind and body. I feel more connected to myself for a while. My mind gets a break from all the tasks with the patient’ (*Respondent 21*). Nurses also described how this emotional connection enhanced their professional pride: ‘I am proud to be able to offer live music and feel the impact it has on the patient. It brings spirit, energy, and motivation to keep fighting. It unites the family around something beautiful that isn't about care or treatment’ (*respondent 26*). This sense of pride was echoed by another nurse: ‘I think it's great to go to work on a Thursday and know, oh yes – it's today that the music is coming. That's just a bonus in the book’ (*Informant 4*). Nurses emphasised not only how it increased their sense of professional pride but also how it transformed an ordinary workday into a source of genuine joy and fulfilment. The anticipation of music served as a welcome counterbalance to the inherent stresses of clinical work, enriching the professional experience. Furthermore, the nurses found that it enhanced the quality of nursing care: ‘I believe it enhances the quality of nursing – being able to address the whole person and offer live music’ (*Informant 7*).

### 5.6 The Nurse as a Gatekeeper Shielding the Patient

The nurses' gatekeeping role emerged as a key aspect of their care practice. They attentively evaluated patients' physical and emotional readiness, sometimes choosing to withhold music to prevent overload or fatigue. This reflected a nuanced, patient‐centred application of clinical judgement, prioritising individual well‐being over the generalised use of the intervention. Although not directly addressed in the survey, this role emerged clearly throughout interviews and open‐ended survey responses. Nurses described thoughtfully evaluating when live music was appropriate, aware of the potential risk of overstimulation in vulnerable patients: ‘Sometimes we say no to music on behalf of the patients because we think they might not be able to handle it’ (*Informant 1*). Another nurse elaborated: ‘Some patients should not have too many stimuli, so we choose to forgo the music on their behalf, out of consideration for them’ (*Informant 3*). These decisions were not expressions of doubt in the value of music but rather measured responses to each patient's specific needs and condition. Patients' limited energy was also considered: ‘Some patients don't have the energy to have live music in their room on top of everything else they have to deal with during the day. They are too tired to be present with a musician and would often rather just sleep’ (*Respondent 20*).

### 5.7 Integration of Quantitative and Qualitative Results

Our results show that patient‐tailored live music supports nurses' emotional well‐being, facilitates restorative breaks and enhances professional fulfilment and nursing care quality. Qualitative insights illustrate how music creates meaningful moments, fosters engagement and allows nurses to act as patient‐centred gatekeepers, while quantitative ratings confirm consistent agreement on its positive impact and meaningfulness. Together, these data highlight how music both enriches staff experience and strengthens patient‐centred care. Table 4 presents a joint display of the integrated quantitative and qualitative results.

**TABLE 4:** A joint display of quantitative and qualitative findings.

Category	Quantitative results (survey); mean and SD	Qualitative findings (survey & interviews)	Integration/interpretation
An emotional breath of fresh air	Positive experience: *M* = 1.39, (SD = 0.59); Personally meaningful: *M* = 1.56, (SD = 0.75)	Music evokes strong emotions, reflection and vulnerability.	Live music provides emotional relief and supports nurses' well‐being.
An intense and meaningful atmosphere	Positive impact on nurse–patient relationship: *M* = 2.37, (SD = 0.82); Positive experience: *M* = 1.39, (SD = 0.59)	Music creates a meaningful, calming atmosphere, enabling presence and human connection.	Supports holistic care and emotional engagement.
Bringing people together in a break from sickness and illness	Positive impact on nurse–patient relationship: *M* = 2.37, (SD = 0.82); Creates meaningful moments for patient–relatives: *M* = 1.66, (SD = 0.74)	Music unites patients, families and nurses, offering emotional connection and shared experiences.	Facilitates meaningful interactions and restorative breaks from clinical stress.
Bringing spirit, energy and motivation into care	Meaningful to nursing care: *M* = 1.82, (SD = 0.79); quality improvement: *M* = 1.74, (SD = 0.79); Improves workday: *M* = 1.91, (SD = 0.84)	Music reduces stress, increases pride, motivation and joy in care.	Enhances nursing care quality and professional fulfilment.
The nurse as a gatekeeper shielding the patient	Not assessed	Nurses observe patient readiness, occasionally withholding music to prevent overload.	Demonstrates patient‐centred judgement complementing music's benefits.

## 6 Discussion

Our findings suggest that live music supports nurses' well‐being by providing emotional release and opportunities for reflection and connection within the demands of clinical work. These experiences were described as deeply moving, sometimes evoking unexpected emotional reactions. Feng et al. similarly found that an 8‐week music therapy programme improved emotional resilience and well‐being, although their participants were not healthcare professionals and took part in structured therapeutic sessions [29].

Compared to this, the nurses in our study experienced live music spontaneously and within their everyday work environment. This suggests that even brief and informal musical experiences can provide emotional relief and support resilience. However, such effects may be short‐lived, indicating that ongoing or structured initiatives might be needed to achieve lasting benefits for nurses' well‐being—consistent with the literature on the emotional burden and stress faced by ICU nurses [6, 7, 8, 9].

Additionally, music was perceived as creating a meaningful and calming atmosphere, allowing nurses to feel like whole individuals rather than just professionals performing a task. This reinforces the idea that music can serve as a holistic intervention, contributing not only to patient‐centred care but also to caregiver well‐being [30]. The sense of gratitude and presence expressed by nurses underscores the value of integrating humanistic elements into clinical settings, supporting broader efforts to improve healthcare environments. This resonates with Herholdt–Lomholdt's concept of the *aesthetic aspects of excellence in nursing*, describing how moments of deep meaning and presence emerge in care situations—what she terms ‘an overflow of meaning.’ These aesthetic experiences, though often invisible, are sensibly felt and may strengthen the relational and existential dimensions of nursing [31]. In this light, live music in clinical settings may serve as a catalyst for these moments, enhancing both the atmosphere and the sense of connectedness between caregivers and patients.

A key category emerging from the findings was the unifying potential of live music. Nurses described how music brought families together during times of crisis, fostering shared experiences that extended beyond the confines of illness and medical care. This aligns with a scoping review showing that healthcare professionals valued music interventions for their capacity to support not only the emotional needs of hospitalised children but also to strengthen the connection between patients, families and staff [32]. Our findings add to this understanding, suggesting that live music—especially when delivered by trained and responsive musicians—can create a shared emotional space where patients, families and caregivers momentarily transcend the clinical context and reconnect as human beings.

Beyond these immediate effects, several nurses described how live music enhanced their sense of professional pride and motivation. They emphasised that music reduced stress, improved the quality of their work and fostered renewed energy in their daily routines. Our findings align with those of a qualitative study conducted in an oncology setting, which likewise showed that musical interventions created calming and emotionally restorative moments for staff, enhancing well‐being and connection [33]. A main distinction lies in the context: whereas that study was carried out in communal hospital areas with shared experiences, our research took place in the ICU, where nurses acted as clinical gatekeepers to protect vulnerable patients. This underscores how context shapes both the experience and practical application of music within healthcare. Together, these results suggest that live music can strengthen nurses' sense of meaning and engagement over time, potentially mitigating burnout and supporting long‐term emotional health. Recognising and nurturing these positive effects may help maintain motivation and commitment, enabling nurses to provide high‐quality, compassionate care [6, 7, 8, 9].

In our results, live music was also described as an add‐on to nurses' professional practice, a perspective also supported by the research project Meaningful Music in Health Care (MiMiC), where health professionals viewed live music as a positive addition that created moments of peace, pleasure or new connection with patients [34]. These findings highlight live music's dual role as both a unifying force and a meaningful source of professional pride. By fostering emotional connection and shared experiences, music not only supports patients and families but also contributes to nurses' well‐being, motivation and sense of purpose in their clinical practice. This reflects what de Wit describes as a cultural shift in healthcare—from task‐centredness towards relationship‐focused, person‐centred care—where presence, human connection and meaning are increasingly recognised as core components of good clinical practice [35]. However, while live music was generally perceived as beneficial, its implementation also raised questions about clinical judgement and patient sensitivity. Our qualitative findings identified challenges related to nurses acting as gatekeepers and deciding when to allow or withhold music based on their assessment of the patient's condition. Concerns about overstimulation raise an important ethical and clinical consideration: how to balance the potential benefits of music with the need to protect vulnerable patients from excessive sensory input.

The category of nurses acting as gatekeepers can also be understood in light of the Danish ICU context, where many patients are awake and communicative during treatment. As Lærkner et al. describe, caring for conscious intensive care patients involves continuous interpretation of subtle verbal and non‐verbal cues, as patients may experience fear, confusion or sensory overload [36]. In this setting, the nurse's gatekeeping role extends beyond clinical assessment to include a relational and ethical dimension—balancing protection and stimulation while remaining responsive to the patient's cues. This resonates with the principles of PCC described in our background, where respect for patients' values, needs and preferences forms the foundation for interaction [13]. When applied in this context, live music may act as both a facilitator and a test of PCC: it can open space for meaningful, human connection with the awake patient, yet it also demands heightened attentiveness to individual tolerance and emotional readiness. Thus, live music becomes part of the same ethical negotiation that defines high‐quality, person‐centred intensive care.

This careful negotiation between stimulation and protection echoes the concept of shielding, which captures how nurses intuitively safeguard patients from cognitive strain [37]. By fostering calm, respectful and structured environments, nurses aim to preserve patients' dignity amidst disorientation and critical illness—an approach that aligns with their gatekeeping role in determining when music may support, or potentially overwhelm, the individual patient.

Our findings underscore the multidimensional value of live music in intensive care, not only as a patient‐centred intervention but also as a resource for emotional resilience, professional meaning and human connection among nurses. While its implementation requires attentiveness to individual needs and clinical context, live music emerges as a subtle yet powerful contributor to more compassionate and holistic care environments.

### 6.1 Limitations

A central strength of this study lies in its convergent mixed‐methods design, which enabled a nuanced understanding of ICU nurses' experiences by combining quantitative trends with qualitative depth. The high survey response rate (72%) strengthens the representativeness of the findings, and the use of validated tools, including face‐ and content‐validated questionnaires and established interview and analysis frameworks, supports the study's methodological rigour. While the high response rate supports robustness, the lack of reliability testing of the questionnaire may limit the generalisability of the quantitative findings. The combination of survey and interview data also allowed for triangulation, enhancing the credibility and richness of the results. Moreover, the study addresses a notable gap in the literature by focusing on the effects of live music interventions on nurses in adult ICU settings.

Certain limitations should, however, be acknowledged. The interview sample was small (*n* = 7) and drawn from a single hospital setting, which may constrain the transferability of the qualitative findings. Member checking of interviews was not conducted, which may limit the credibility of the results. The risk of social desirability bias must also be considered, as participants may have offered more favourable responses—consciously or unconsciously—to align with what they perceived to be the interviewer's expectations or to support the intervention.

### 6.2 Implications and Recommendations

This study highlights live music as a feasible and meaningful addition to ICU care, with benefits extending beyond patient well‐being to include nurse resilience, motivation and professional pride.

Integrating music interventions into everyday practice may help create more humanised and emotionally supportive environments. We recommend close collaboration between nurses and musicians in the planning and delivery of music. For critical care nurses, this implies that live music may serve as a complementary tool to reduce stress, strengthen nurse–patient–family relationships and enhance holistic nursing practice, while remaining attentive to individual patient needs and potential risks of overstimulation.

Implementing music interventions in ICU settings not only relies on individual engagement but also on supportive organisational conditions. Leadership endorsement and clear institutional frameworks are crucial for ensuring that such initiatives are prioritised, resourced and integrated into routine care. Future research should further explore how such interventions can be integrated sustainably, identifying structural enablers and barriers and evaluating long‐term outcomes to support both patient well‐being and the emotional health of healthcare professionals across critical care and other clinical environments.

## 7 Conclusion

This study contributes to the growing body of evidence supporting the integration of arts‐based interventions in healthcare by demonstrating that ICU nurses perceive live music as a valuable, person‐centred, and professionally enriching practice. The findings indicate that live music may enhance emotional well‐being, foster a sense of presence and strengthen relational aspects of care in high‐intensity environments. Furthermore, the intervention was associated with enhanced professional pride and greater job satisfaction. While implementation requires clinical judgement and contextual sensitivity, it additionally depends on organisational support to ensure sustainable integration. Live music thus emerges not only as a therapeutic complement to holistic care but also as an aesthetic and humanising element in nursing practice.

## Funding

The study was supported by the Novo Nordisk Fonden (NNF), Grant number NFF0087330.

## Ethics Statement

According to Danish law and The Danish Healthcare Act § 42, interviews and survey studies of this kind require no formal ethical approval. The project was reviewed and registered on 1 May 2023, in the Internal List of Research Projects in the Central Denmark Region under case number 1‐16‐02‐404‐25. The department heads of the participating ICUs approved the study. Participation in the survey was considered to imply consent. All participants provided both oral and written consent. Participants were informed of the voluntary nature of their participation, confidentiality measures and their right to withdraw at any time. The study was conducted in accordance with the Declaration of Helsinki.

## Consent

The authors have nothing to report.

## Conflicts of Interest

The authors declare no conflicts of interest.

## Supporting information


**Table S1:** Good reporting of a mixed‐methods study (GRAMMS) checklist.

## Data Availability

The data that support the findings of this study are available from the corresponding author, LT, upon reasonable request.
