# Supplementary material for: ‘Nurses’ Assessment and Perception of Live Music in the Intensive Care Unit: A Convergent Mixed‐Methods Study
Source: Nurs Crit Care. 2026 Feb 9;31(2):e70356. doi: 10.1111/nicc.70356 (PMC12914499; doi:10.1111/nicc.70356)
Supplement: Supplementary file 1 — Table S1: Good reporting of a mixed‐methods study (GRAMMS) checklist. [file NICC-31-0-s001.docx]

Table S1. Good Reporting of A Mixed Methods Study (GRAMMS) checklist

To ensure transparency in the reporting of the study: *Nurses’ perception of live music in the intensive care unit: A convergent mixed methods study.*

| **Guideline** | **Section: page** |
| --- | --- |
| Describe the justification for using a mixed methods approach to the research question | Design and methods pg. 4 |
| Describe the design in terms of the purpose, priority and sequence of methods | Aim pg. 4  Design and methods pg. 4  The music intervention pg. 4 |
| Describe each method in terms of sampling, data collection and analysis | Setting and participants pg. 5  Data collection pg. 5  Data analysis pg. 6 |
| Describe where integration has occurred, how it has occurred and who has participated in it | Data analysis pg. 6  Results pg. 7-13  Discussion pg. 13-16 |
| Describe any limitation of one method associated with the present of the other method | Discussion pg. 13-16  Limitations pg. 16 |
| Describe any insights gained from mixing or integrating methods | Discussion: pg. 13-16  Limitations pg. 16 |

O'Cathain A, Murphy E, Nicholl J. The quality of mixed methods studies in health services research. J Health Serv Res Policy. 2008;13: 92-98.
